# A decrease in serum creatinine after ICU admission is associated with increased mortality

**DOI:** 10.1371/journal.pone.0183156

**Published:** 2017-08-24

**Authors:** Hye Ran Kang, Si Nae Lee, Yun Ju Cho, Jin Seok Jeon, Hyunjin Noh, Dong Cheol Han, Suyeon Park, Soon Hyo Kwon

**Affiliations:** 1 Division of Nephrology, Soonchunhyang University Hospital, Seoul, Korea; 2 Hyonam Kidney Laboratory, Soonchunhyang University Hospital, Seoul, Korea; 3 Department of Biostatistics, Soonchunhyang University Hospital, Seoul, Korea; University of Sao Paulo Medical School, BRAZIL

## Abstract

**Background:**

The elevation of serum creatinine (SCr), acute kidney injury (AKI), is associated with an increase of mortality in critically ill patients. However, it is uncertain whether a decrease in SCr in the intensive care unit (ICU) has an effect on outcomes.

**Methods:**

In a retrospective study, we enrolled 486 patients who had been admitted to an urban tertiary center ICU between Jan 2014 and Dec 2014. The effect of changes in SCr after ICU admission on 90 day mortality was analyzed. Patients were classified into 3 groups based on change in SCr after ICU admission: a stable SCr group (Δ SCr < 0.3mg/dL during ICU stay), a decreased SCr group (Δ SCr ≥ -0.3 mg/dL during ICU stay) and an increased SCr group with criteria based on the KDIGO AKI criteria.

**Results:**

In total, 486 patients were identified. SCr decreased in 123 (25.3%) patients after ICU admission. AKI developed in 125 (24.4%) patients. The overall 90-day mortality rate was 29.0%. In a Kaplan-Meyer analysis, the mortality of the AKI group was higher than that of other groups (p<0.0001). Patients with a decrease in SCr had a higher mortality rate than those with stable SCr (p<0.0001). A Cox analysis showed that both a decrease in SCR (HR, 3.56; 95% CI, 1.59–7.97; p = 0.002) and an increase in SCr (AKI stage 1, HR, 9.35; 95% CI, 4.18–20.9; p<0.0001; AKI stage 2, HR, 11.82; 95% CI, 3.85–36.28; p<0.0001; AKI stage 3, HR, 17.41; 95% CI, 5.50–55.04; p<0.0001) were independent risk factors for death compared to stable SCr.

**Conclusion:**

Not only an increase in SCr, but also a decrease in SCr was associated with mortality in critically ill patients.

## Introduction

The elevation of serum creatinine (SCr), acute kidney injury (AKI), is associated with an increase of mortality in critically ill patients [1–4]. Mortality rates of patients with AKI in intensive care units (ICUs) range from 20.8% to 49% [1, 2, 5, 6]. Patients with even one episode of mild AKI have significantly lower long-term survival rates than critically ill patients with no AKI [3]. Also, the severity of AKI is associated with increased mortality in critically ill patients [1, 2, 7–9].

Studies have evaluated the effect of a decrease in SCr on clinical outcome in various settings. One study showed that a profound decrease in SCr (Δ-0.3 mg/dl) was associated with increased mortality in patients when compared to no change in SCr after cardiothoracic surgery [10]. However, another report revealed no significant difference in mortality between a decrease in SCr and stable SCr among patients with ST-segment elevation myocardial infarction undergoing primary percutaneous coronary intervention (PCI) [11]. In addition, another study concluded a decrease in SCr in the first 24hours after cardiac arrest was indicative of good prognosis [12]. These prior studies were in specific populations, such as those with cardiac surgery, PCI and cardiac arrest. The effect of a decrease in SCr in critically ill patients after ICU admission is uncertain. Augmented renal clearance (ARC), defined as creatinine clearance exceeding 130mL/min per 1.73m^2^, is a common phenomenon in septic patients of ICUs [13]. The early phase of sepsis is often associated with a hypermetabolic condition leading to renal creatinine clearance without improved kidney function [14]. In critically ill patients, ARC at ICU admission is associated with increased mortality [15]. Antimicrobial drug treatment for infection has more therapeutic failure in patients with ARC than those without ARC [16]. ARC may lead to lower blood concentrations of drugs that are cleared by the kidneys, and the potential need for increased drug doses in critically ill patients [13, 15, 17, 18]. However, the definition of ARC does not include change in SCr during ICU admission. Critical illness with or without AKI in the ICU is also associated with significant falls in SCr that persist to hospital discharge [19]. This suggests that a decrease in SCr after ICU admission has clinical implications.

We conducted this study to determine whether a decrease of SCr affected clinical outcomes in patients after ICU admission and compared that to AKI and stable SCr.

## Materials and methods

### Study design

We performed a retrospective observational study examining the effects of changes in SCr in patients who were admitted to the ICU at Soonchunhyang University Seoul Hospital between January 1 and December 31, 2014. This hospital is a tertiary care center and has 750 beds, including 2 adult ICU units with a combined total of 50 beds. The study protocol was approved by the institutional review board (IRB) of Soonchunhyang hospital and conducted in accordance with the Declaration of Helsinki. This was an anonymous observational study, the need for informed consent was waived (IRB no: 2016-02-018-001). Patients were excluded if they were admitted for less than 48 hours, were younger than 18 years, had a do not resuscitate order, had history of renal replacement therapy or end-stage renal disease, or had a kidney transplant.

### Data collection

For each patient, Acute Physiology and Chronic Health Evaluations II (APACHE II) scores were calculated during the first 24 hours of the ICU admission. The patients were classified into three groups based on change in SCr after ICU admission: (1) a stable SCr group (Δ SCr < 0.3mg/dL during ICU stay); (2) a decreased SCr group (Δ SCr ≥ -0.3 mg/dL during ICU stay); and (3) an increased SCr group with criteria based on Kidney Disease: Improving Global Outcomes (KDIGO) AKI criteria [20]. Baseline SCr was defined as the first SCr measurement on ICU admission. Our ICU policy is to access the kidney function at least 4 times a week. Thus, SCr was checked every morning in 95% of patients. After admission, SCr was measured by clinical indications. Any patient with an AKI episode was placed in the AKI groups and patients requiring dialysis after ICU admission were placed in the AKI group. The SCr of patients in the stable group did not vary more than 0.3 mg/dL during the duration of their ICU stay. Demographic information; principle admission diagnosis (including trauma, sepsis, respiratory failure without sepsis, post-surgery, others); comorbidities (including malignancy, chronic kidney disease, diabetes, liver cirrhosis, hypertension, cerebrovascular accident, coronary artery disease, heart failure, chronic obstructive pulmonary disease) were obtained from medical records by ICU staffs admission notes, not previous laboratory data. Ventilator care; vasopressor/inotropic use; diuretic use; antibiotic use; and laboratory data at admission to ICU (including albumin, bilirubin, blood urea nitrogen (BUN) and SCr, were obtained from electronic medical records. SCr was determined using the Jaffe reaction with a HITACHI 7600–110 autoanalyzer (Hitachi, Tokyo, Japan). For SCr measurement, the intra-series precision was not evaluated during the study period, but the interassay CV can be presented because it is internal quality controlled with QC material daily. The low CV values for 2014 were mean 0.60 mg/dL, CV 3.97%, CV high value mean 6.60 mg/dL, CV 0.94%. Our clinical laboratory has been monitored by Korean Association of Clinical survey and Quality Management. The amount of fluids given to the patients were assessed for three days after admission to determine if there was a dilution effect on SCr. Fluid were classified as “Chloride-liberal” fluids, which contained supraphysiological concentrations of chloride (0.9% saline, 20% and 5% albumin) or ‘Chloride-restrictive’ fluids, which contained chloride concentrations closer to plasma (0.45% saline, Ringer’s lactate).

### Outcome data

The primary outcome of study was all cause death up to 90 days. Secondary outcomes were length of stay (LOS) in the ICU, LOS in the hospital and in-hospital mortality. The LOS was defined as the number of days between the date of admission and the date of discharge or death. All enrolled patients were included in the analyses for all cause death up to 90 days, LOS in the ICU, LOS in the hospital, and in-hospital mortality.

### Statistical analyses

The demographic details, comorbidities, and laboratory and outcome data were analyzed. Continuous data are presented as mean ± standard deviation (SD) or median [interquartile range], and nominal data are presented as percentages. Comparisons among multiple groups were performed using the χ^2^ test or the Fisher exact test for nominal variables, and the Kruskal-Wallis test was used for numerical variables where appropriate. Kaplan-Meier cumulative survival curves were plotted for each group. The log-rank test was used to examine differences in the survival curves among the groups. The mortality was adjusted according to the Cox proportional hazards model. All factors that were significant at p<0.2 in the univariate analysis were considered for the multivariate analysis. Stepwise selection was used to select the best subset of predictors in a risk prediction model. All statistical analyses were performed using SPSS 14.0 (SPSS, Chicago, IL, USA). A 2-sided P<0.05 was considered to be statistically significant.

## Results

### Characteristics

**Fig 1:**
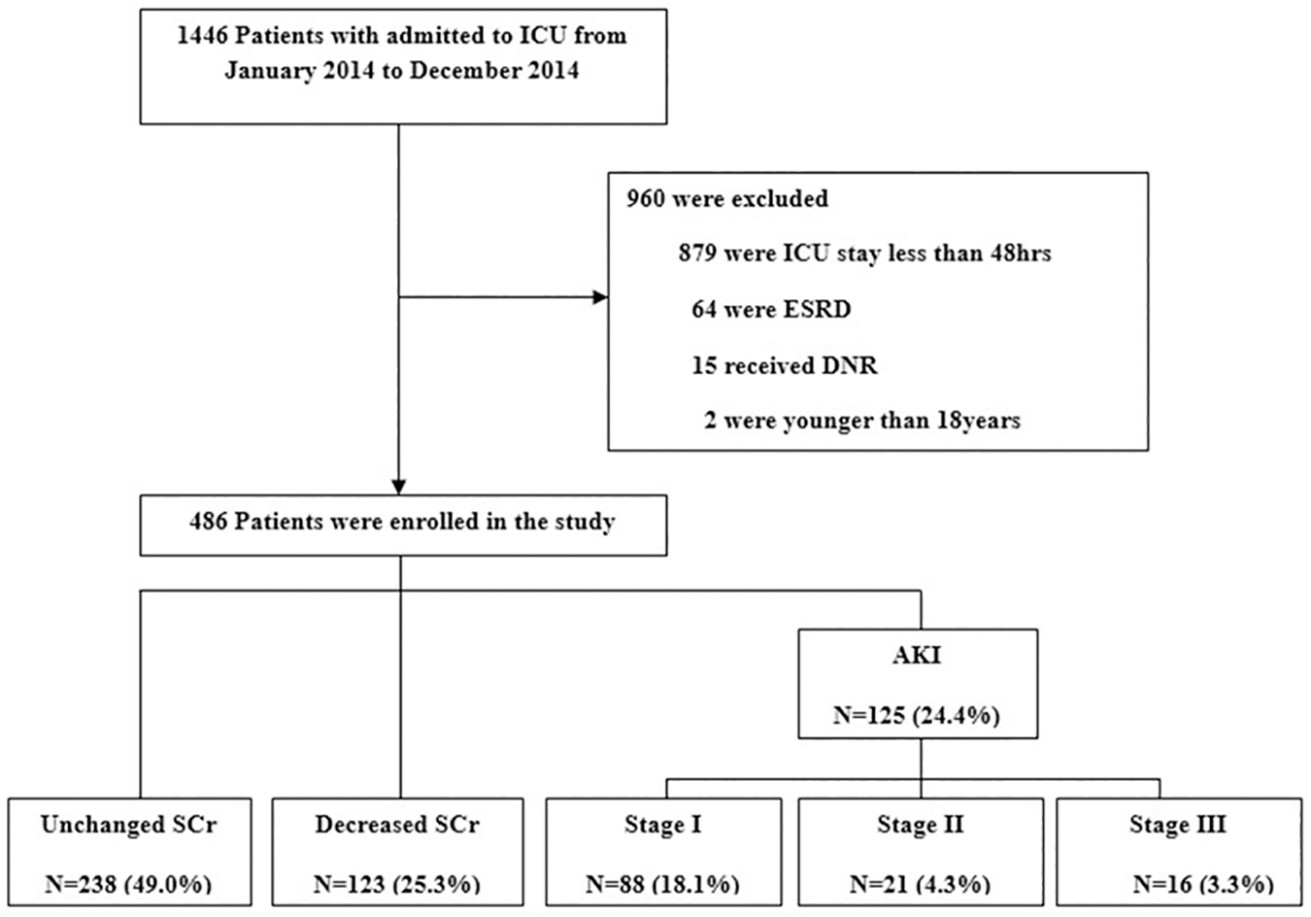
Flowchart of patients included in the study. ICU, intensive care unit; ESRD, end-stage renal disease; DNR, do not resuscitate; AKI, acute kidney injury; SCr, serum creatinine.

During the study period, a total of 1,446 patients were admitted to the ICU at Soonchunhyang University Hospital. After exclusion, 486 patients were included for analysis (Fig 1). The patients whose ICU stay was less than 48hrs were mostly patients of drug intoxication and post-surgery recovery for monitoring. Most of these patients were transferred to the general ward within one or two days.

The mean age of the patients was 65.7±17.7 years with a male predominance of 59.9%. SCr decreased in 123 (25.3%) patients after ICU admission. AKI developed in 125 (24.4%) patients based on KDIGO AKI criteria. Among patients with AKI, 88 (18.1%) were classified as stage1, 21 (4.3%) as stage 2; and 16 (3.3%) as stage3. Two hundred thirty eight (49.0%) patients met the criteria for the stable creatinine group (Fig 1). Characteristics of the enrolled patients, including initial laboratory data, are shown in Table 1 according to change in SCr.

**Table 1:** Baseline characteristics of patients according to serum creatinine changes.

Variable	Stable SCr (n = 238)	Decreased SCr (n = 123)	AKI stage 1 (n = 88)	AKI stage 2 (n = 21)	AKI stage 3 (n = 16)	P value
Age (y), mean ± SD	64.0 ± 18.3	67.5 ± 17.9	67.3 ± 16.7	64.5 ± 8.2	69.1 ± 11.9	0.317
Sex (male, n (%))	135 (56.7)	77 (62.6)	56 (63.3)	13 (31.9)	10 (62.5)	0.742
Height (cm), mean ± SD	162.1 ± 9.4	163.0 ± 10.0	162.2 ± 9.38	161.0 ± 8.2	168.2 ± 9.5	0.190
Weight (kg), mean ± SD	61.0 ± 13.8	61.1 ± 14.1	61.6 ± 13.6	55.7 ± 11.1	67.7 ± 13.2	0.116
BMI(kg/m^2^), mean ± SD	23.2 ± 4.1	23.0 ± 4.9	23.4 ± 4.5	21.4 ± 3.4	23.8 ± 3.4	0.307
APACHE II score, mean ± SD	12.1 ± 5.6	15.3 ± 6.2	18.0 ± 6.8	20.1 ± 8.0	23.1 ± 4.7	<0.0001
Admission type						
Trauma, n (%)	23 (9.7)	11 (8.9)	2 (2.3)	2 (9.5)	0 (0.0)	0.029
Sepsis, n (%)	46 (19.3)	41 (33.3)	33 (37.5)	6 (28.6)	5 (31.3)	0.001
Respiratory failure without Sepsis, n (%)	30 (12.6)	20 (16.3)	21 (23.9)	7 (33.3)	8 (50.0)	0.002
Post-surgery, n (%)	28 (11.8)	10 (8.1)	6 (6.8)	1 (4.8)	1 (6.3)	0.127
Other, n(%)	106 (44.5)	39 (31.7)	23 (26.1)	5 (23.8)	2 (12.5)	0.001
Admission via ER, n (%)	187 (78.6)	100 (81.3)	55 (62.5)	12 (57.1)	6 (40.0)	<0.0001
Comorbidity						
Malignancy, n (%)	39 (16.4)	18 (14.6)	20 (22.7)	9 (42.9)	3 (18.8)	0.040
CKD, n (%)	5 (2.1)	17 (13.8)	8 (9.1)	1 (4.8)	1 (6.3)	0.047
Diabetes, n (%)	43 (18.1)	43 (35.0)	39 (44.3)	10 (47.6)	6 (37.5)	<0.0001
Liver cirrhosis, n (%)	22 (9.2)	12 (9.8)	9 (10.2)	4 (19.0)	0 (0.0)	0.622
Hypertension, n (%)	83 (34.9)	50 (40.7)	43 (48.9)	5 (23.8)	8 (50.0)	0.055
CVA history, n (%)	28 (11.8)	10 (8.1)	13 (14.8)	1 (4.8)	3 (3.3)	0.540
Coronary artery disease, n(%)	15 (6.3)	15 (12.2)	13 (14.8)	3 (14.3)	2 (4.2)	0.017
Ventilator care, n (%)	64 (26.9)	34 (27.6)	41 (46.6)	10 (47.6)	12 (75.0)	<0.0001
Vasopressor/inotropics, n (%)	42 (17.6)	45 (36.6)	46 (52.3)	14 (66.7)	9 (56.3)	<0.0001
Diuretics, n (%)	85 (35.7)	59 (48.0)	69 (78.4)	11 (52.4)	11 (68.8)	<0.0001
Antibiotics, n (%)	144 (60.5)	98 (79.7)	76 (86.4)	16 (76.2)	13 (81.3)	<.0001
Laboratory data						
WBC (/mm^3^)	10901 ± 8885	14298 ± 9404	15546 ± 2147	13180 ± 9161	10400 ± 5683	0.014
Hemoglobin (g/dL)	12.0 ± 2.6	11.8 ± 3.0	11.1 ± 2.7	10.9 ± 2.2	12.4 ± 2.7	0.038
Hematocrit (%)	36.2 ± 7.3	35.4 ± 8.9	33.5 ± 8.0	32.4 ± 6.8	37.2 ± 8.3	0.028
Albumin (g/dL)	3.71 ± 0.81	3.35 ± 0.86	3.10 ± 0.81	3.24 ± 0.78	3.36 ± 0.99	<0.0001
Bilirubin(mg/dL)	0.78 ± 0.65	1.86 ± 4.61	1.86 ± 3.33	1.68 ± 1.88	0.78 ± 0.35	0.004
Total cholesterol (mg/dL)	168.1 ± 55.5	133.1 ± 50.5	139.2 ± 65.1	193.4 ± 68.9	180.8 ± 34.5	<0.0001
Sodium (mmol/L)	138.5 ± 6.2	139.8 ± 7.8	137.5 ± 5.6	135.7 ± 6.0	138.9 ± 5.7	0.031
Potassium (mmol/L)	3.8 ± 0.6	4.3 ± 1.0	4.1 ± 0.8	4.1 ± 1.0	4.3 ± 0.8	<0.0001
Chloride (mmol/L)	101.0 ± 6.9	102.0 ± 9.9	100.5 ± 7.1	98.0 ± 6.2	101.4 ± 6.4	0.236
HCO3^-^(mmol/L)	22.8 ± 4.5	19.3 ± 5.9	20.4 ± 10.8	18.9 ± 5.1	19.8 ± 5.4	<0.0001
BUN (mg/dL)	16.7 ± 9.2	39.1 ± 28.4	26.7 ± 19.5	22.5 ± 13.2	25.2 ± 14.7	<0.0001
Creatinine (mg/dL)	0.78 ± 0.32	1.78 ± 1.18	1.39 ± 1.11	0.94 ± 0.43	1.36 ± 1.06	<0.0001

SCr, serum creatinine; AKI, acute kidney injury; SD, standard deviation; BMI, body mass index; APACHE II, Acute physiology and Chronic Health Evaluations II; CKD, chronic kidney disease; CVA, cerebrovascular accident; WBC, white blood cell

There was a difference in APACHE II scores between the SCr change groups(p<0.0001). The decreased SCr group had more chronic kidney disease than the other groups (p = 0.047) and required more vasopressors/inotropics, diuretics and antibiotics that patients with stable SCr (p<0.0001). Ventilator care and use of vasopressors/inotropics and diuretics in AKI groups were significantly higher than in the other groups (p<0.0001). SCr at ICU admission in the decreased SCr group was higher than in the other groups (p<0.0001). The SCr at ICU admission was 1.78 ± 1.18 mg/dL, and the lowest SCr level was 0.88 ± 0.64 mg/dL in the decreased SCr group. The mean of change amount of SCr in the group decreased SCr after ICU admission was -0.93 ± 0.91 mg/dL. The mean of baseline SCr in the “decreased SCr” group survivors was 1.93 ±1.14 mg/dL, non-survivor is 1.73 ±1.20 mg/dL. There was no significant difference in values of SCr decrease between patients that survived and non-survivors in the decreased SCr group (p = 0.489). The mean of SCr decrease values in the decreased SCr group survivor was -0.90 ± 0.95 mg/dL, and non-survivors was -1.01 ±0.82 mg/dL. There was no difference in the the amount of chloride-liberal fluid administered between the groups. However the amount of chloride-restrictive fluid administered was less in the stable SCr group than the other groups (Table 2). Total fluid replacement for the stable Cr group was lower than the other groups (p = 0.001).

**Table 2:** The amount of fluids given for three days after admission according to serum creatinine change.

	Stable SCr(n = 238)	Decreased SCr(n = 123)	AKI stage 1 (n = 88)	AKI stage 2 (n = 21)	AKI stage 3 (n = 16)	P value
Chloride-liberal fluids (mL) median [range]	1967 (993–2700)	2133 (767–3300)	2208 (939–3108)	1933 (528–2683)	2532 (1569–3508)	0.219
Chloride-restrictive fluids (mL), median [range]	1200 (406–1815)	1389 (393–2167)	1632 (712–2383)	1612 (657–3082)	1633 (857–3731)	<0.0001

### Outcomes

The median total LOS was 32.3 [28–42] days. The ICU LOS for patients in the decreased SCr group was significantly longer than that of the stable SCr group (p<0.0001). The in-hospital mortality in the AKI groups was higher than the other groups. In addition, in-hospital mortality in the decreased SCr group was significantly higher than in the stable SCr group (p<0.0001) (Table 3).

**Table 3:** Outcomes according to serum creatinine change.

	Stable SCr (n = 238)	Decreased SCr (n = 123)	AKI stage 1 (n = 88)	AKI stage 2 (n = 21)	AKI stage 3 (n = 16)	P value
ICU LOS(d), median [range]	7.6 [3.0–9.0]	9.3 [3.0–12.0]	13.3 [5.0–16.0]	11.6 [4.0–15.5]	12.4 [4.0–14.8]	<0.0001
Total LOS(d), median [range]	33.1 [12.0–42.0]	28.5 [11.0–36.0]	32.6 [11.0–49.8]	32.3 [8.5–40.5]	32.8 [7.8–42.8]	0.807
Hospital mortality (%)	19 (8.0)	28 (22.8)	17 (19.3)	14 (66.7)	13 (81.3)	<0.0001

**Fig 2:**
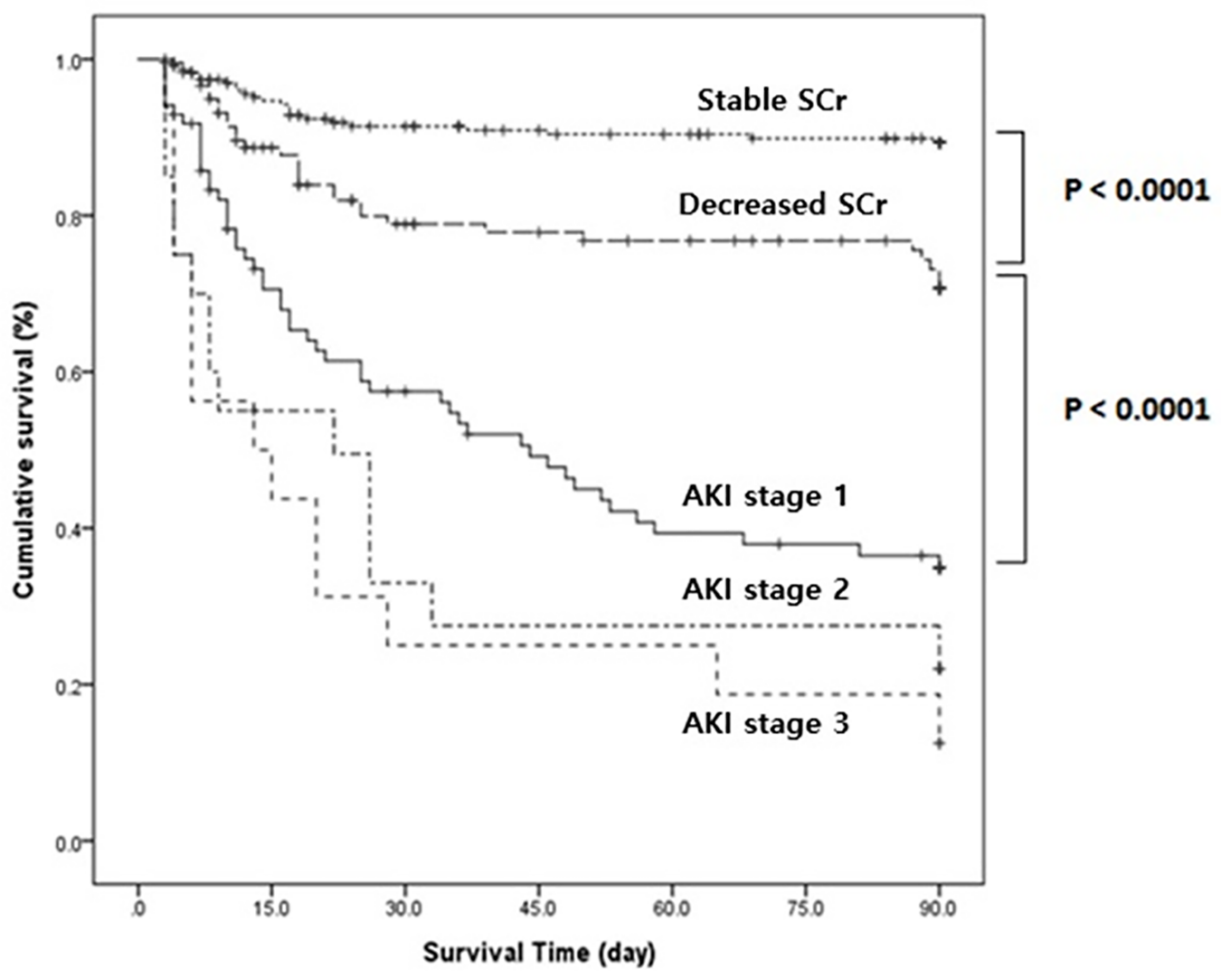
Kaplan-Meier survival curve for the groups.

The overall 90-day mortality was 29.0%. In the Kaplan-Meyer analysis, mortality in the AKI group was higher than in the other groups (p<0.0001). Patients in the decreased SCr group had a higher mortality rate compared to those with stable SCr group (p<0.0001) (Fig 2).

In the multivariate analysis, a decreased in SCr was an independent risk factor for mortality (Table 4).

**Table 4:** Cox-regression analysis of factors associated with mortality in critically ill patients.

	Cox-regression analysis of mortality			
Clinical variables	Univariate analysis		Multivariate analysis	
	Hazard ratio (95% CI)	P value	Hazard ratio (95% CI)	P value
Age	1.03 (1.02–1.04)	<0.0001		
BMI	0.94 (0.90–0.99)	0.012		
APACHE II score	1.13 (1.09–1.17)	<0.0001		
Ventilator care	2.52 (1.79–3.55)	<0.0001		
Vasopressor/inotropics	3.28 (2.33–4.63)	<0.0001	2.23 (1.32–3.76)	0.003
Diuretics	3.21 (2.19–4.70)	<0.0001		
Decrease Cr	2.90 (1.69–5.0)	<0.0001	3.56 (1.59–7.97)	0.002
AKI stage 1	8.45 (5.15–13.87)	<0.0001	9.35 (4.18–20.90)	<0.0001
AKI stage 2	13.46 (7.01–25.86)	<0.0001	11.82 (3.85–36.28)	<0.0001
AKI stage 3	17.28 (8.86–33.69)	<0.0001	17.41 (5.50–55.04)	<0.0001

CI, confidence interval; BMI, body mass index; APACHE II, Acute physiology and Chronic Health Evaluations II

## Discussion

In this study, a change in SCr in any direction after ICU admission had clinical implications in critically ill patients. A decrease in SCr after ICU admission was associated with increased mortality compared to a stable SCr. We found that a decrease in SCr after ICU admission was frequent. The higher mortality in patients with a decrease in SC compared to those with stable SCr at hospital discharge continued to be seen at 90 days follow-up.

The decreased SCr group had differing initial clinical characteristics compared to the other groups. Their baseline SCr was higher than the other groups and they had more CKD. In addition, they needed more vasopressor/inotropic support, diuretics and antibiotics. These findings suggest these patients had a more serious course of disease during their ICU stay. The decreased SCr group had higher APACHE II score than stable group. The APACHE II score has been validated for predicting mortality in the ICU setting [21]. The higher APACHE II score may had have an impact on prognosis in the decreased SCr group. However, after adjusting for APACHE II score, a decrease in SCr still had clinical implications for predicting death in critically ill patients.

In a steady state, a decrease in Cr production or an increase in glomerular filtration rate (GFR) can decrease SCr. However, real improvement in kidney function was not common in critically ill patients [22]. Why did the decreased SCr group have poorer outcomes than the stable SCr group? Although we did not have SCr data before ICU admission for all the patients in the decreased SCr group, the group might have been more likely to have previously developed AKI. Even after recovery, AKI has significant long-term adverse effect on outcomes [23, 24]. The patients in the decreased SCr group may have had a more complicated treatment course. They had more comorbidities, including CKD, diabetes, and coronary artery disease, than the stable SCr group and needed more diuretics and inotropics. Previous studies on mortality in patients with a decrement of SCr had similar findings [10, 25]. Patients had higher preoperative SCr, had more preoperative comorbidities, and were more likely to need diuretics and ionotropic support. Thus, those patients experienced a more serious course in which SCr was more diluted by higher volume replacement and blood transfusions [10]. Creatinine is distributed throughout the total body water volume, and positive fluid balances can be expected to increase total body water. This will have the effect of diluting SCr concentration and providing a falsely lowered serum SCr in the ICU [22]. This is associated with delayed recognition of AKI. It is possible those patients with decreased SCr would be re-classified as having AKI after adjusting for exact fluid balance. In some cases, a rise SCr could be diluted by fluid replacement. In our study, the amount of fluid administered to the decreased SCr group was not different from the AKI groups for the first 3 days after ICU admission. However, we were not able to assess the exact fluid balance, including oral intake, urinary output and insensible losses. In addition, sepsis can decrease production of creatinine, without changes in body weight, hematocrit, or extracellular fluid volume. This could blunt the increase in SCr in AKI with sepsis [26]. Sepsis in patients with increased renal clearance in the ICU is strongly associated with sub-therapeutic antibiotic serum concentration during the initial treatment period [14, 16–18]. It was also possible that the decrease in SCr might have been ignored by the physician. Our ICU policy was to adjust the drug dosing according to GFR. Almost all patients’ antibiotic doses were corrected for estimated GFR. However, this policy may not be enough. Therapeutic drug monitoring (TDM) is the method of choice to optimize dosing in patients with a change of kidney function [17]. TDM was not performed for all patients receiving medication, so sub-therapeutic antimicrobial drug dosing cannot be determined with our data. However, the decreased SCr group received more antibiotic treatment than the stable SCr group. Also, a retrospective study examined a specific population that initially presented with increased SCr on hospital admission and had a subsequent decrease in SCr of 0.3 mg/dL or greater than normal within 48 hours in the general hospital setting [25]. This group had poorer outcomes than patients with stable SCr but better hospital mortality than patients with AKI, even in those with fully reversing AKI. These results were similar to our results. Fluctuating kidney function should be considered a potential confounder in optimal antibiotic drug therapy. In addition, nutrition status can affect prognosis in the ICU setting. It is well known that malnutrition is associated with increased GFR and poor outcomes [27]. Critical illness is associated with significant falls in SCr from admission to discharge because of a decrease in creatinine production resulting from the loss of muscle mass potentially combined with reduced hepatic creatinine production and dietary changes [19].

It is surprising that a decrease in SCr after ICU admission was common. Previous AKI studies did not distinguish this group from the non-AKI group. It is possible doing so would have lowered the proportion of the patients with stable SCr and findings on the outcomes for patients with stable SCr would have been better. Among postoperative patients and patients in the cardiac intensive unit, just 5% and 3.5% respectively had a decrease in SCr [10, 11]. These patients represent a specific population with heart problems. However, our cohort included a variety of different patients receiving active treatment. Our study demonstrates that a decrease in SCr is more common in the ICU setting than in postoperative care or coronary care unit. Several prospective studies reported that more than 50% of ICU patients experienced high renal clearance after ICU admission [13], which was associated with mortality [16]. A significant number of critically ill patients had fluctuating renal function. However, the studies measured 24 hour urinary Cr clearance and the patients had higher absolute levels of renal clearance. In our study, we measured changes in SCr after ICU admission. This population would be different than those with ARC.

Our study had several limitations. First, in all single-center studies, the results should be validated in other settings. Second, because of the study’s retrospective nature, it was possible that all of the important outcome variables were not included in the multivariate analysis. Third, GFR was not measured in this study. Thus, we don’t know how much changes in SCr represent changes in renal function in critically ill patients. Fourth, data regarding the precise amount of fluids given and changes in muscle mass and protein intake were not recorded in the majority of patients. Therefore we cannot elucidate their possible effects on SCr. Fifth, we did not consider urinary output in our cohort, so it is possible that some cases of AKI were not assigned well. But many studies of AKI did not include urinary output. Finally, the criteria for decreased SCr (Δ SCr ≥ -0.3 mg/dL during ICU stay) seem to be arbitrary and needs more scientific research. However, this definition was used in previous studies [10, 11] and a change of SCr > 0.3 mg/dL was assumed to be significant in AKI definition. In addition, Jaffe reaction with a HITACHI autoanalyzer could be affected by the some drugs such as rifampicin and levodopa in an exceptional case. This study did not consider this effect.

## Conclusions

In conclusion, we demonstrated that not only an increase in SCr, but also a decrease in SCr is associated with increased mortality in critically ill patients. Decrement of SCr seems to be related to complicated clinical situation. Our findings have a potentially important clinical implication, suggesting the clinicians should pay attention to any subtle change in SCr after ICU admission. Whether this consideration of this change of SCr at ICU may help to optimize therapies and improve outcomes need further clinical study.

## Supporting information

S1 Table Original individual data. (XLSX)
